# Cellulitis Caused by Pseudomonas putida: A Case Report and Review of the Literature

**DOI:** 10.7759/cureus.85317

**Published:** 2025-06-04

**Authors:** Masakazu Kakurai, Yoshihiro Moriyama

**Affiliations:** 1 Dermatology, Tsuchiura Kyodo General Hospital, Tsuchiura, JPN

**Keywords:** cellulitis, pseudomonas putida, pseudomonas species, septic shock, skin and soft tissue infections

## Abstract

*Pseudomonas putida* is a gram-negative bacterium often found in environments like water and soil. It is typically linked to hospital-acquired infections, particularly in people who have medical devices or catheters. The bacterium rarely causes skin and soft tissue infections (SSTIs). Herein, we present a case of cellulitis in the right lower extremity caused by *P. putida*. A 78-year-old Japanese man with a history of nephrotic syndrome due to membranous nephropathy, treated with oral prednisolone and cyclosporine, presented with painful swelling, warmth, and purpura in the right lower extremity. The exploratory incision findings excluded necrotizing soft tissue infections. Therefore, cellulitis was diagnosed, and intravenous meropenem hydrate was initiated. Because of persistent hypotension despite fluid therapy, the patient was transferred to the intensive care unit for vasopressor support. From day 2 to day 3, the patient underwent direct hemoperfusion with a polymyxin B immobilized fiber column to treat septic shock. On day 3, the patient’s vital signs and painful swelling had improved. Subsequently, two sets of blood cultures and a wound culture yielded *P. putida *alone. As the patient’s general condition and laboratory data had improved, meropenem hydrate was discontinued on day 11. In our literature review, we found that SSTIs, including cellulitis, caused by *P. putida* tend to occur in the lower extremities of older adults with multimorbidity or immunosuppression, and sepsis may be associated with poor prognosis.

## Introduction

*Pseudomonas putida*, a gram-negative, rod-shaped bacterium commonly found in water and soil habitats, was previously thought to have low pathogenicity [1]. However, the bacterium is increasingly being considered a significant human pathogen [1]. Because *P. putida* can colonize moist and inanimate hospital surfaces, it causes nosocomial infections with poor prognosis, especially in patients with medical devices or catheters and in immunocompromised patients [1]. In contrast, *P. putida *rarely causes skin and soft tissue infections (SSTIs), including cellulitis, and most reported cases are community-acquired infections with a favorable prognosis [2]. Because of rarity, clinical characteristics of *P. putida *SSTIs remain largely unknown. For adding characteristics to existing reports, we present a case of *P. putida *cellulitis and review 14 previously reported cases (including ours) of *P. putida *SSTIs.

## Case presentation

**Figure 1:**
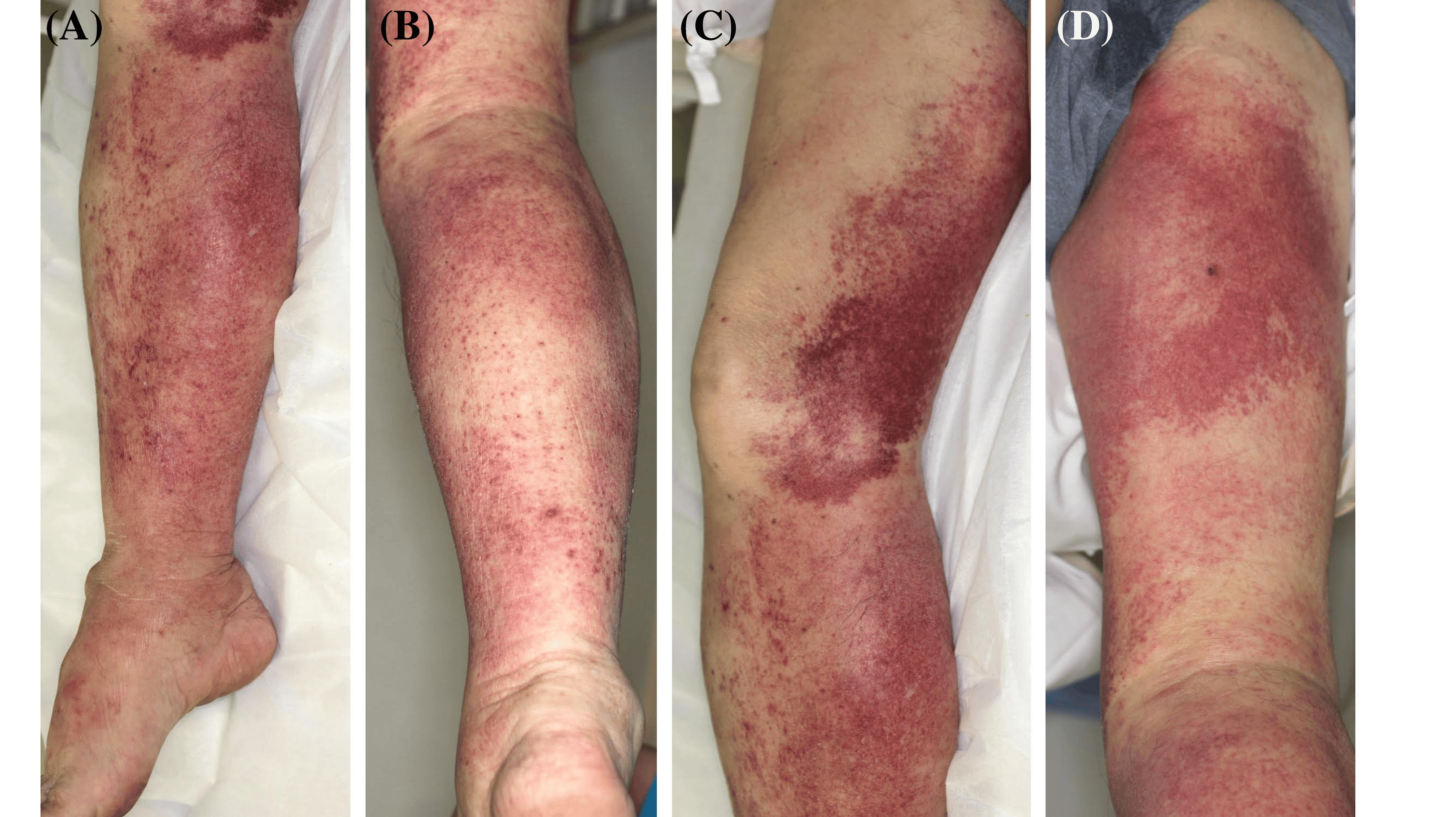
Skin manifestations (A-D) Painful swelling and warmth with punctate purpura tending to coalesce in the right lower extremity: (A) Median aspect of the right lower leg. (B) Posterior aspect of the right lower leg. (C) Median aspect of the right thigh. (D) Posterior aspect of the right thigh.

A 78-year-old Japanese man presented to the emergency department with right lower extremity pain for the previous three days. His medical history included nephrotic syndrome due to membranous nephropathy, diagnosed two months prior to this visit. Despite treatment with oral prednisolone (PSL) at 25 mg daily and cyclosporine at 100 mg daily, the disease control was poor. On admission, the patient had an axillary body temperature of 37.1°C, a heart rate of 91 beats per minute, a blood pressure of 84/53 mmHg, and a respiratory rate of 21 breaths per minute, without impaired consciousness. Physical examination revealed painful swelling and warmth with punctate purpura tending to coalesce in the right lower extremity (Figure 1A-1D). Skin lesions were limited to this region without the inclusion of wounds or skin ulcers.

**Figure 2:**
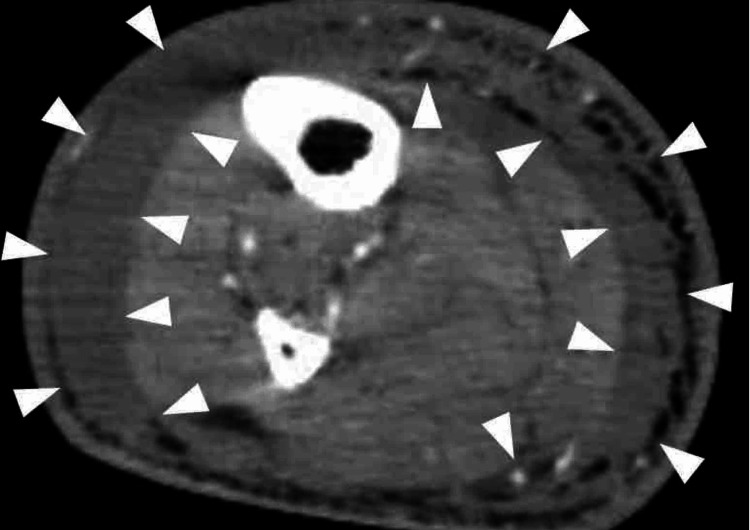
Computed tomographic findings Swelling of the right lower extremity with fluid above the superficial fascia (indicated by the white arrows). The image shows the right lower leg.

Computed tomographic images showed swelling of the right lower extremity, accompanied by the presence of fluid above the superficial fascia without abnormal gas (Figure 2). Based on the imaging, no infection in other regions was suspected.

Blood tests revealed a white blood cell (WBC) count of 6,660/μL, blood urea nitrogen of 33 mg/dL, creatinine of 1.16 mg/dL, and C-reactive protein level of 13.37 mg/dL (Table 1). A leukocyte esterase dipstick test was negative, and the number of WBCs in the urine was within normal range (0-4 WBCs/HPF).

**Table 1:** Laboratory data of blood samples

Blood test	Reference value (male)	On arrival	On day 10
White blood cell (/μL)	3,300-8,600	6,660	5,840
Neutrophils (%)	38.0-74.0	78	87
Hemoglobin (g/dL)	13.5-17.0	10.7	11.8
Platelet (×10^4^/μL)	15.0-35.0	12.8	16.8
Aspartate aminotransferase (U/L)	13-30	30	26
Alanine aminotransferase (U/L)	10-42	62	46
Lactate dehydrogenase (U/L)	124-222	561	475
Sodium (mEq/L)	138-145	132	136
Potassium (mEq/L)	3.6-4.8	3	4.1
Urea nitrogen (mg/dL)	8-20	33	23
Creatinine (mg/dL)	0.65-1.07	1.16	0.57
Creatine kinase (U/L)	59-248	340	-
C-reactive protein (mg/dL)	0-0.14	13.37	0.84
Hemoglobin A1c (%)	4.6-6.2	7.2	-
Glucose (mg/dL)	70-109	151	-
Procalcitonin (ng/mL)	0-0.50	1.01	-
β-D-glucan (pg/mL)	<5.0	<5.0	-

**Figure 3:**
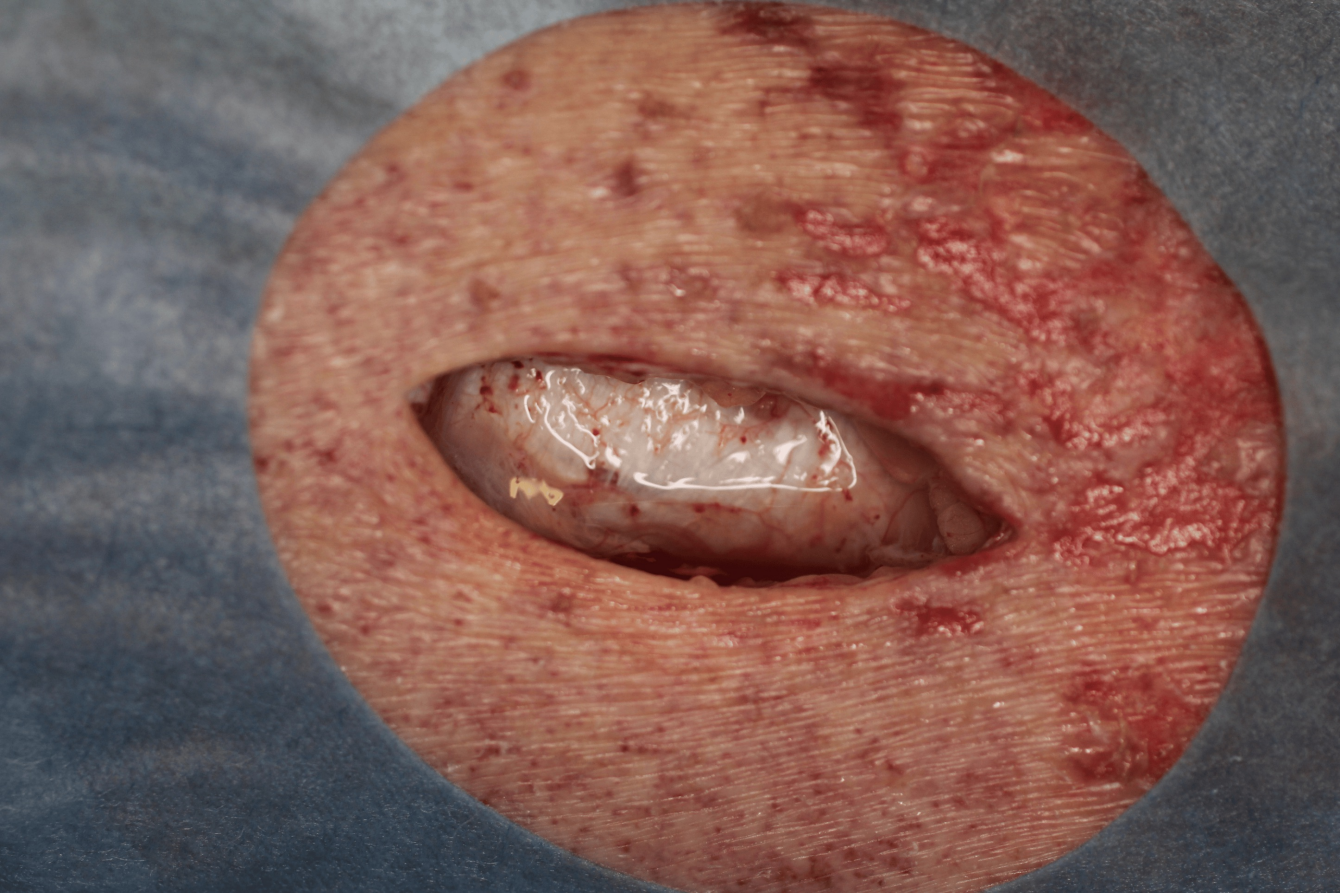
Exploratory incision findings There is no evidence of soft tissue necrosis, abscess, or odor.

As the patient complained of severe pain in the right lower extremity and had purpura, an exploratory incision was made to a depth above the superficial fascia of the right lower leg. No soft tissue necrosis or abscesses were observed (Figure 3).

Taken together, cellulitis was diagnosed, and intravenous meropenem hydrate was initiated. Because of persistent hypotension despite fluid therapy, the patient was transferred to the intensive care unit for vasopressor support. On day 2, two sets of blood cultures taken from the artery upon admission grew gram-negative bacilli. From day 2 to day 3, the patient underwent direct hemoperfusion with a polymyxin B immobilized fiber column to treat septic shock. On day 3, the patient’s vital signs and painful swelling had improved. Subsequently, two sets of blood cultures and a wound culture yielded *P. putida* alone (the antimicrobial susceptibility of the isolate is shown in Table 2). On day 11, as the patient’s general condition and laboratory data had improved, meropenem hydrate was discontinued.

**Table 2:** Antimicrobial susceptibility of the Pseudomonas putida obtained from blood and exudate cultures Susceptibility testing was performed using the MicroScan WalkAway system (DxM 1096). MIC: minimum inhibitory concentration; R: resistant; S: susceptible

Antimicrobial agent	MIC (μg/mL)	MIC interpretation
Cefepime	≤2	S
Piperacillin-tazobactam	≤8	S
Meropenem	≤2	S
Imipenem	≤1	S
Levofloxacin	≤0.5	S
Sulfamethoxazole-trimethoprim	>2	R

## Discussion

*P. putida*, formerly considered a bacterium with low pathogenicity, is increasingly identified as a significant human pathogen [1]. The bacterium generally causes nosocomial infections in patients with medical devices or catheters and in immunocompromised patients [1]. In a previous study of 18 patients with nosocomial *P. putida *bacteremia, the central venous catheter was the common primary site of infection, followed by ventilator-associated pneumonia, and 14 patients (77%) had device-related infections (e.g., central venous catheter, ventilator, endotracheal tube, biliary stent, and indwelling urinary catheter) [1]. Furthermore, 56% of the patients were in an immunocompromised state (e.g., solid tumor, hematologic malignancy, and liver cirrhosis) [1].

Although rare, *P. putida *causes SSTIs, including cellulitis, wound infection, soft tissue infection, and necrotizing fasciitis. In a previous review of 55 patients with *P. putida *infections at a medical center in Taiwan, SSTIs represented only three patients (5%) [3]. In a systematic review that examined blood cultures from patients with cellulitis, of 1,578 patients with blood cultures, 125 (7.9%) were positive, and 35 of 125 (28%) involved gram-negative bacteria [4]. The most common gram-negative bacteria were *Pseudomonas aeruginosa *and *Escherichia coli*, which were detected in six patients, respectively [4]. Although *P. putida *was not reported in this systematic review, five patients with *P. putida *cellulitis, including ours, have been reported to date [2,5-7]. In addition, to our knowledge, 14 patients with *P. putida *SSTIs have been reported (five with cellulitis, four each with wound and soft tissue infection, and one with necrotizing fasciitis) (Table 3) [2,3,5-13].

**Table 3:** Reports of SSTIs caused by Pseudomonas putida AF: atrial fibrillation; AIH: autoimmune hepatitis; AUD: alcohol use disorder; AZA: azathioprine; CKD: chronic kidney disease; DM: diabetes mellitus; HLD: hyperlipidemia; HT: hypertension; PSL: prednisolone; PVD: peripheral vascular disease; SLE: systemic lupus erythematosus; SSTIs: skin and soft tissue infections

Author and year	Age and sex	Diagnosis	Medical history	Lesion site	Bacteremia	Surgery	Course
Ono et al. (2024) [2]	70/F	Cellulitis	AIH (PSL 30mg, AZA 50mg), DM	Left lower leg	Yes	No	Survived
Yang et al. (1996) [3]	NA	Wound infection	NA	NA	NA	Yes	Survived
Yang et al. (1996) [3]	NA	Wound infection	NA	NA	NA	Yes	Survived
Yang et al. (1996) [3]	NA	Wound infection	NA	NA	NA	Yes	Survived
Hayashi et al. (2020) [5]	51/F	Cellulitis	SLE (PSL 7mg)	Left lower leg	Yes	No	Survived
Salabei et al. (2020) [6]	75/M	Cellulitis	CKD	Right lower extremity	Yes	No	Survived
Rowe et al. (2025) [7]	63/M	Cellulitis	CKD, HT, DM, HLD	Right lower extremity	Yes	No	Survived
Chen et al. (2005) [8]	78/F	Soft tissue infection	None	Lower legs	Yes	No	Survived
Carpenter et al. (2008) [9]	24/M	Wound infection	None	Right lower leg	No	Yes	Survived
Thomas et al. (2013) [10]	80/F	Soft tissue infection	CKD, PVD, malnutrition	Left lower extremity	Yes (septic shock)	No	Deceased
Hardjo Lugito et al. (2015) [11]	51/F	Soft tissue infection	HT, DM	Right foot	NA	Yes	Survived
Singh et al. (2021) [12]	57/F	Necrotizing fasciitis	HT, HLD	Right lower extremity	Yes (septic shock)	Yes	Deceased
El Hasbani et al. (2023) [13]	71/M	Soft tissue infection	HT, HLD, AF, AUD	Left lower extremity	Yes	No	Survived
Our case	78/M	Cellulitis	Nephrotic syndrome (PSL 25mg, cyclosporine 100mg), DM	Right lower extremity	Yes (septic shock)	No	Survived

In our literature review, SSTIs mainly occurred in older adults (n = 11; mean age, 63 years), with no gender predominance [2,5-13]. Among the patients with available information, nine of 11 patients (82%) had underlying conditions, such as diabetes mellitus, chronic kidney disease, and peripheral vascular disease [2,5-7,10-13], suggesting these as risk factors for *P. putida *SSTIs. Three of the 11 patients (27%) received immunosuppressive therapy, including PSL, cyclosporine, and azathioprine [2,5]. In all 11 patients with available data, the lower extremities were affected [2,5-13]. Nine of the 10 patients (90%) who underwent blood culture testing had bacteremia [2,5-8,10,12,13], whereas the remaining one (10%) with wound infection had negative blood cultures [9]. Nine of the 14 patients (64%) had wounds or skin ulcers with signs of infection [2,3,6,7,9-11], and three (21%) were exposed to water before the onset of SSTIs [6,8,12], suggesting that the skin is generally the primary site of infection rather than hematogenous dissemination due to bacteremia. The present case had no history of wounds, skin ulcers, or exposure to contaminated water or soils; however, since no infectious lesions other than the skin were found during the initial visit, *P. putida* may have directly invaded the skin, resulting in cellulitis followed by septic shock. In summary, *P. putida *SSTIs tend to develop in the lower extremities of older adults with multimorbidity or immunosuppression.

*P. putida *has higher antimicrobial susceptibility than *P. aeruginosa *[1,11]. However, carbapenem-resistant and multidrug-resistant (MDR) *P. putida *have recently become a concern [1,11]. Among 18 patients with nosocomial *P. putida *infections, four (22%) and five (28%) isolates were resistant to imipenem and meropenem, respectively [1]. MDR strains were found in 28% of *P. putida *isolates [1]. Furthermore, the 30-day mortality rate was 39%; carbapenem-resistant *P. putida *was associated with high mortality rates [1]. In contrast, among *P. putida* SSTIs for which antimicrobial susceptibility results were available, only one patient was resistant to carbapenem (MDR *P. putida*) [11]. *P. putida *SSTIs are generally caused by community-acquired infections [2]; however, because carbapenem-resistant and MDR *P. putida *can be isolated in cases of SSTIs, appropriate antibiotic therapy and nosocomial infection control measures, including personal protective equipment, are important. Our literature review revealed that of nine patients who developed bacteremia [2,5-8,10,12,13], three developed septic shock, two of whom died [9,12]. Therefore, although *P. putida *SSTIs presenting with bacteremia have a good prognosis, if they progress to sepsis, the prognosis may be poor. The present case developed septic shock, but the early use of appropriate antibiotics and polymyxin B hemoperfusion may have contributed to a favorable outcome [14].

The limitations of this study include the restrictive inclusion criteria for English language publications, a limited number of cases, and missing data in several studies. Further accumulation of cases of *P. putida* SSTIs is required to clarify their epidemiology, clinical characteristics, and appropriate treatments to reduce mortality.

## Conclusions

The present case is unique in that the patient developed cellulitis due to *P. putida*, an unusual cellulitis pathogen. To our knowledge, 14 patients with *P. putida *SSTIs have been reported. In our literature review, *P. putida *SSTIs typically occur in the lower extremities of older adults with multimorbidity or immunosuppression, and sepsis may be associated with poor prognosis. Gathering additional cases of *P. putida *SSTIs is necessary to advance the understanding of epidemiology and clinical characteristics and optimize appropriate treatment strategies.
